# A Case of Rare Fourth Branchial Arch Anomaly: Presentation and Surgical Management

**DOI:** 10.7759/cureus.73987

**Published:** 2024-11-19

**Authors:** Yewen Qin, Shelly Ling

**Affiliations:** 1 Otolaryngology Department, Southampton General Hospital NHS Foundation Trust, Southampton, GBR; 2 Emergency Department, Salisbury NHS District Hospital, Salisbury, GBR

**Keywords:** branchial arch malformation, branchial cyst, embryology, fourth branchial anomaly, recurrent neck abscess

## Abstract

This report presents a case of a six-year-old male patient with recurrent left-sided neck abscesses who presented four times over a span of two years. At each presentation, the child had developed left-sided neck swelling, pain, and fevers that required hospital admission. In the patient’s most recent admission in 2020, a fourth branchial cleft anomaly was confirmed on CT, and the patient was taken to the operation theatre for ultrasound-guided aspiration and cauterisation of the sinus fistula tract under direct pharyngoscopy.

This allowed for definitive management of the area and, to date, has prevented any recurrence. Although fourth branchial anomalies are rare within overall branchial arch pathologies, this case highlights the importance of considering this to be a differential diagnosis in a patient presenting with recurrent neck abscesses, particularly in children.

## Introduction

The branchial embryonic system consists of six arches of paired mesodermal origin; these are separated by clefts formed of ectoderm and pouches formed of endoderm. From the fourth branchial pouch are derived the laryngeal cartilages, superior laryngeal nerve, left thoracic aorta, right subclavian artery, laryngeal and pharyngeal constrictor muscles, the thyroid interfollicular cells, and the superior parathyroid glands [1]. The fourth branchial arch sinuses commence in the pyriform fossa and exit the larynx close to the cricothyroid joint and behind the thyroid gland, travelling superficial to the recurrent laryngeal and hypoglossal nerves [2]. In the presence of a sinus fistula/tract, these would continue either along the trachea and oesophagus and around the aorta if left-sided and around the subclavian artery if right-sided [3,4].

Fourth branchial cleft cysts are rare congenital anomalies that account for 2-4% of branchial cleft malformation pathologies [5]. When present, they can be mistaken for other diagnoses such as thyroiditis and lymphatic swellings. They are usually diagnosed in childhood with recurrent neck swelling and infections, but this is not a definitive sign. Left-sided anomalies vastly outnumber right-sided ones, presenting at 97% (left-sided), and females are marginally more affected than males [3,6]. In this report, we describe the case of a recurrent fourth branchial cleft anomaly presentation in a paediatric patient and how this was surgically managed using drainage and cauterisation to close the sinus.

## Case presentation

The patient initially presented in November 2018 when he was four years old with a left-sided neck swelling and coryzal symptoms. During that admission, the patient was treated with intravenous clavulanic acid-amoxicillin with good response. The patient had three further admissions in May, September, and December 2020 due to similar recurrent left-sided neck swelling, pain, and fever, requiring intravenous antibiotics as well as ultrasound-guided aspiration in his initial admission in 2018. He was assessed in the inpatient and outpatient settings and underwent CT in 2018 and ultrasound in 2019 and 2020, as well as barium swallow. The imaging at the time did not report any obvious malformations and was suggestive of potential thyroid origin. However, the differential diagnosis of a fourth branchial cleft abnormality was also put forward, and thus plans were made to assess and electively manage this. Due to the onset of the COVID-19 pandemic, the patient’s work-up was temporarily withheld as local outpatient clinics and elective operations were restricted from March 2020 as the United Kingdom was put into lockdown status.

**Figure 1:**
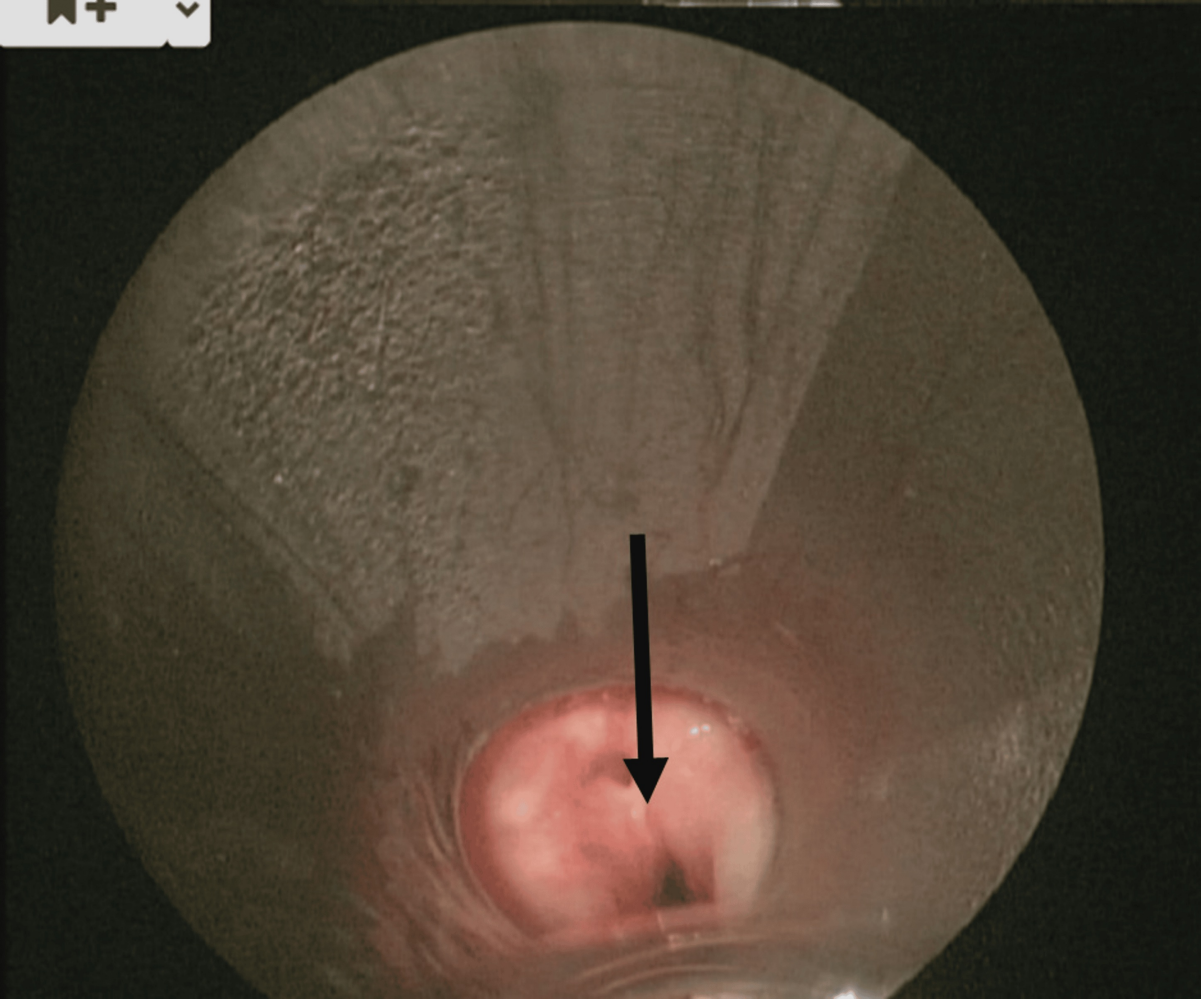
Endoscopic image of the fourth branchial cleft anomaly prior to procedures, with the arrow indicating sinus opening.

**Figure 2:**
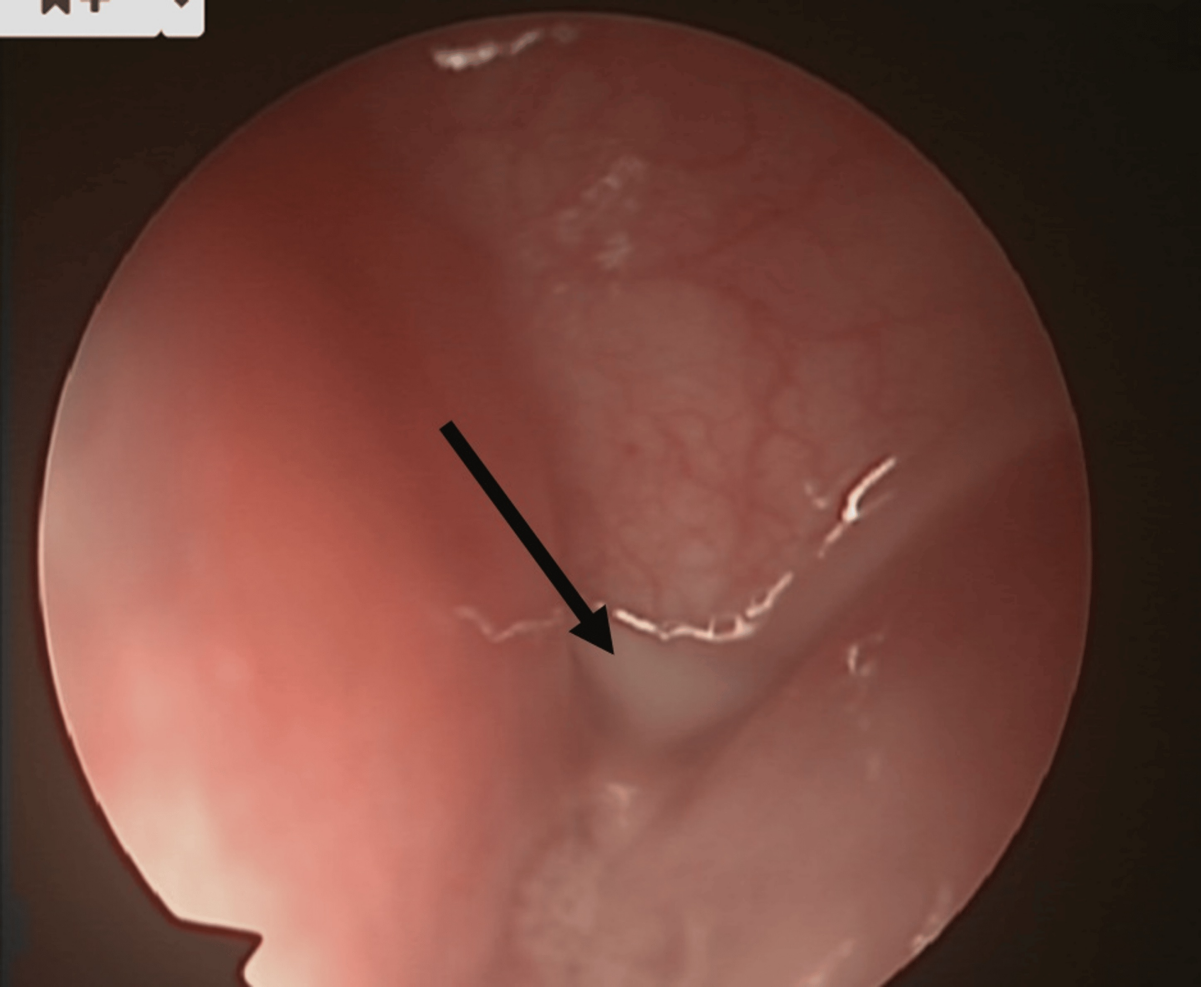
Endoscopic image of discharging pus (arrow) from the fourth branchial cleft cyst anomaly.

**Figure 3:**
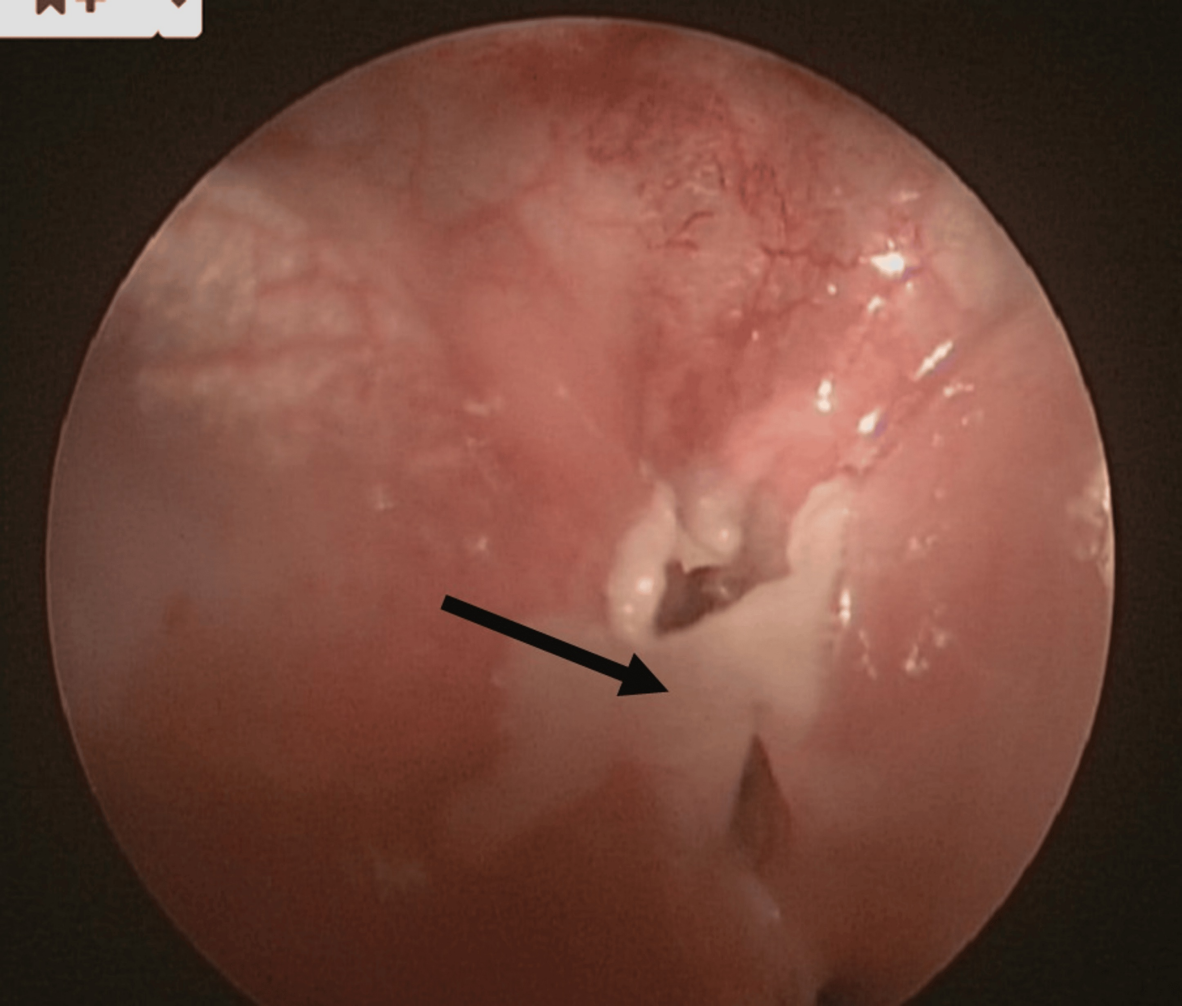
Endoscopic image post-cauterisation (arrow) of the fourth branchial cleft anomaly.

In December 2020, at six years of age, the same patient presented again to the emergency department with left-sided neck swelling, fever, and worsening neck pain. During this admission, a CT scan was repeated, which reported a multiloculated collection approximately 33 x 30 x 36 mm in size within the left lower neck surrounding the lateral aspect of the left lobe of the thyroid. There was posterior extension towards the oesophagus/hypopharynx, and anteriorly the anomaly was less than 1 cm from the skin surface. The report stated that a fourth branchial cleft cyst was to be considered as a strong differential diagnosis. Given these findings, the patient was taken for ultrasound-guided aspiration and pharyngoscopy under general anaesthetic to identify the fourth branchial cleft cyst. This was done in a stepwise manner; firstly, the abscess was drained under ultrasound guidance from the cavity close to the inferior aspect of the thyroid medially towards the carotid; this removed approximately 8 mL of purulent fluid. Then, pharyngoscopy was performed, which identified the cyst (Figure 1) and the discharging fistula connecting to the left piriform fossa (Figure 2). This area was cauterised with ball monothermy (Figure 3). The patient was admitted for further three days to complete the IV antibiotic course before being discharged. As of writing, the patient has been stable with no further reported recurrence.

## Discussion

Fourth branchial cleft cysts are rare, but they often cause recurrent neck infections, and therefore definitive management is advisable. This reduces the need for repeated courses of antibiotics, particularly as these patients are young. Thus, the confirmation of a diagnosis and definitive management is favourable.

In this case, the patient was known to have an underlying predisposing pathology and was undergoing work-up in an outpatient setting. The acute presentation of left-sided neck pain, swelling, and fever precluded this process and allowed for operative intervention at an earlier stage. In other literature, it has been advised to attain radiological confirmation of the branchial cleft cyst/sinus prior to surgical intervention. There are various surgical approaches that have been utilised, including cauterisation [7]. Open surgical approaches to excise the branchial cleft anomaly have also been described once any acute infection has settled [8]. In our case, the discharging sinus was utilised as an indicator for the procedure, and intra-operative ultrasound-guided aspiration was performed in the same operation theatre prior to the cauterisation. This allowed for direct visualisation of the sinus/fistula tract, and therefore the cauterisation could be directed accurately.

With regards to the preferable management of such cases, a balance needs to be drawn between endoscopic approaches, such as electrocauterisation, and open surgery, such as neck resection and hemithyroidectomy, regarding risks and benefits.

Meta-analysis data indicate that incision and drainage of fourth branchial cleft cysts in paediatric patients have high levels of recurrence of up to 89%, and these cases eventually require either open surgery or endoscopic cauterisation to control definitively; as such, direct incision and drainage does not present a satisfactory option for definitive management [9]. This was also reflected in our case as the patient underwent drainage in 2018 prior to his surgery.

In the electrocauterisation method, the region is directly visualised using laryngoscopy or pharyngoscopy and then any pus or discharge from the anomaly is drained; finally, directed pulses of electrocautery are applied to seal the anomaly using an insulated electrocautery device with a ball or needlepoint attachment to ensure adequate contact whilst minimising external thermal injury risk [10]. A progression on this technique includes using a balloon catheter to dilate the sinus prior to electrocauterisation to further visualise the sinus more clearly [7].

Open surgery mainly consists of excision of the sinus with or without partial thyroidectomy. The sinus tract is confirmed prior to surgery via barium swallow or CT/MRI if possible. The surgical approach is incision via the cricoid cartilage with progressive dissection of the muscle layers until the sinus tract is reached at the cricothyroid joint. Once the tract has been identified, it can be resected towards the pyriform apex; due to the proximity to the thyroid gland, the surgeon can decide to perform a hemithyroidectomy at this stage in order to remove any potential fourth cleft tissue within the gland along with the sinus tract [11,12]. However, the sinus tract has been reported to have various anatomical variations which complicates open surgery. In addition, the recurrent laryngeal nerve presents a potential site for injury throughout [13]. Open neck resections have been reported in adult cases to provide good outcomes after confirmed diagnosis and therefore been suggested as the definitive option [12,14].

Interestingly, there is a recent publication on a combination approach of surgery which involves a first stage of cauterisation of the sinus tract before proceeding to an open resection of the now-cauterised tract, again with inclusion of hemithyroidectomy. This technique was reported to have an 100% surgical cure rate with minimal complications on follow-up [15]. As this is a novel procedural method put forward, it shows that there are ongoing developments in the management of fourth branchial cleft sinuses.

However, longer-term data for paediatric patients have shown endoscopic electrocauterisation as a viable alternative as it negates the risks associated with open surgical dissection and subsequent scar tissue formation that can be significant in paediatric cases, whilst also providing good long-term outcomes [7,16]. The latter approach is also what has been illustrated in our case report, as the patient’s post-operative recovery was uneventful and he has also not presented again with a recurrence of the neck abscess or other post-operative complications.

## Conclusions

This case highlights how fourth branchial cleft anomalies can present with recurrent neck infections and swellings in paediatric patients and that this diagnosis may be initially overlooked due to the rarity of presentations. In our patient, a fourth branchial cleft anomaly was already being considered as the diagnosis with corresponding work-up, and the coincidental re-presentation of the neck swelling expedited this process for definitive management. The encouraging post-operative situation and current results also highlight that endoscopic cauterisation of these anomalies can produce promising outcomes in a young patient without having to resort to larger-scale open surgery and its associated risks.
